# Cenospheres-Reinforced PA-12 Composite: Preparation, Physicochemical Properties, and Soaking Tests

**DOI:** 10.3390/polym14122332

**Published:** 2022-06-09

**Authors:** Damian S. Nakonieczny, Magdalena Antonowicz, Thomas Heim, Andrzej S. Swinarew, Paweł Nuckowski, Krzysztof Matus, Marcin Lemanowicz

**Affiliations:** 1 Institute for Manufacturing Technologies of Ceramic Components and Composites, University of Stuttgart, 70569 Stuttgart, Germany; thomas.heim@ifkb.uni-stuttgart.de; 2 Department of Biomedical Engineering, Silesian University of Technology, Akademicka 2A, 44-100 Gliwice, Poland; magdalena.antonowicz@polsl.pl; 3 Faculty of Science and Technology, University of Silesia in Katowice, 41-500 Chorzów, Poland; andrzej.swinarew@us.edu.pl; 4 Institute of Sport Science, The Jerzy Kukuczka Academy of Physical Education, 40-065 Katowice, Poland; 5 Materials Research Laboratory, Faculty of Mechanical Engineering, Silesian University of Technology, Konarskiego 18 A, 44-100 Gliwice, Poland; pawel.nuckowski@polsl.pl (P.N.); krzysztof.matus@polsl.pl (K.M.); 6 Faculty of Chemistry, Department of Chemical Engineering and Process Design, Silesian University of Technology, Akademicka 2A, 44-100 Gliwice, Poland; marcin.lemanowicz@polsl.pl

**Keywords:** polyamide PA-12, cenospheres, composites, surface modification, Piranha solution, APTES

## Abstract

The main aim of this research was the preparation of a polymer–ceramic composite with PA-12 as the polymer matrix and modified aluminosilicate cenospheres (CSs) as the ceramic filler. The CSs were subjected to an early purification and cleaning process, which was also taken as a second objective. The CSs were surface modified by a two-step process: (1) etching in Piranha solution and (2) silanization in 3-aminopropyltriethoxysilane. The composite was made for 3D printing by FDM. Raw and modified CSs and a composite with PA-12 were subjected to the following tests: surface development including pores (BET), real density (HP), chemical composition and morphology (SEM/EDS, FTIR), grain analysis (PSD), phase composition (XRD), hardness (HV), and static tensile tests. The composites were subjected to soaking under simulated body fluid (SBF) conditions in artificial saliva for 14, 21, and 29 days. Compared to pure PA-12, PA-12_CS had generally better mechanical properties and was more resistant to SBF at elevated temperatures and soaking times. These results showed this material has potential for use in biomedical applications. These results also showed the necessity of developing a kinetic aging model for aging in different liquids to verify the true value of this material.

## 1. Introduction

Polymer–ceramic composites (PCCs) are increasingly used in biomaterials engineering as functional layers, structural materials, and degradable elements in the human body [1,2]. Due to their potential for replacing metals and their alloys, much hope is placed on PCCs for structural applications [3] and most attention has focused on composites with polymer matrixes of PEEK, PET, PA, and PU [4,5]. PEEK is a good alternative to ceramics and some metal alloys, especially when used in dental prosthetics for fixed prosthetic frameworks or generally fixed prostheses [6]. However, it suffers from significant costs and problems during manufacturing, i.e., high injection temperatures and high unit prices [7]. PA-12 is an alternative to PEEK for applications that do not require significant mechanical strength, i.e., temporary prostheses, frameworks in prosthetics, and CNC-machined inlay–onlay crowns. PA-12 is used increasingly in additive manufacturing and allows extensive processing for additive manufacturing [8,9,10].

PCCs are filled with different ceramics, such as ZrO_2_, TiO_2_, and their various combinations [11,12]. In medical applications, ZrO_2_ and SiO_2_ are the most popular materials, especially for dental applications, i.e., light-cured composites for restorations and fillers for PMMA for CNC systems [13,14]. Ceramic fillers (CFs) are used to increase the mechanical properties of polymers, i.e., tribological wear resistance and tensile strength, or to decrease absorbability [15,16]. Aluminosilicate cenospheres (CSs) are an alternative for the currently used oxide CFs, which are receiving increasing recognition as a filler for PCCs [17,18]. CSs arise from the combustion of hard coal during conventional electricity generation in coal-supplied power plants. They are the most valuable residue in fly ash produced from the combustion of stone coal [19]. It is estimated that the combustion of hard coal annually produces around 10 million tonnes of CSs worldwide, which is approx. 1.5 wt.% of the annual global production of ash from coal [18,20]. CSs are hollow bodies with desirable engineering properties, such as hardness, low bulk density, and complete chemical inertness, which allow them to be used in biomedical engineering applications [19,20]. Taking into account their chemical composition, CSs do not differ significantly from fly ash [19,20]. SiO_2_ and Al_2_O_3_ in different proportions account for more than 90 wt.% of the cenospheres’ composition. CSs can be used as a CF in medical applications because of their low price, the availability of raw materials, good mechanical properties, and no negative impact on living organisms. Attention should be paid to the large variations in chemical compositions, especially the mineral impurities found in the coal and the additives used to improve combustion efficiency and minimize the amount of ash; therefore, these raw materials should be standardized [19,20].

The key issue in PCCs is to form a good adhesive bond between the ceramic and polymer matrix. This bonding can be realized mechanically by obtaining the maximum filler surface development or by chemical modification [15]. The bonding between a CF and polymer matrix can occur via different mechanisms [21,22]: (1) the short-range physical effects of van der Waals forces or (2) the covalent and ionic chemical bonds. Chemical methods that find applications for surface modification include sol–gel methods, layered double hydroxide (LDH), and chemical etching [15,23,24]. Chemical etching and silanization methods have reproducible results and relatively simple processes. These methods aim to functionalize the ceramic surface with functional groups and coupling agents, i.e., -OH, -CH_2_HN_2_, -NH_2_, -CF_3_, -COOH, H_3_PO_4_, aminosilanes, and fluorosilanes [25,26]. CF surface modification is crucial for composites because it helps to prevent unfavorable phenomena from occurring in these materials [27,28]: (1) the formation of a filler gradient in the composite and non-homogeneous distribution of the filler in the matrix; (2) the lack of consistent mechanical properties; (3) surface defects, i.e., pores; and (4) the formation of closed pores. The mechanism to achieve improved bonding in a PCC involves preventing significant differences between the surface energy of the hydrophilic ceramic and that of the hydrophobic polymer matrix. This can lead to the agglomeration and poor dispersion of ceramic filler particles, resulting in the formation of voids and interfacial defects [28]. Coupling agents used for silanization include [28,29,30,31]: (3-aminopropyl)triethoxysilane (APTES), trimethylchlorosilane (TMCS), and (3-mercaptopropyl) trimethoxysilane (MPTMS). Of these, only APTES, whose organic chain contains a functional group capable of forming H-bonds with polymers, provides composites with better thermo-mechanical stability, highlighting the importance of creating stronger interfacial bonds versus a simple surface energy reduction [26].

More attention is being paid to additive manufacturing (AM) methods for the preparation of materials, which, in principle, enables them to approach the properties of materials produced using traditional methods such as injection molding, casting, or milling. According to the international standardization arrangements of ISO and ASTM, (additive manufacturing) AM can be classified into seven subtypes [9]: (1) material extrusion; (2) vat polymerization; (3) binder jetting; (4) material jetting; (5) direct energy deposition; (6) sheet lamination; and (7) powder bed fusion (PBF). The first group of methods includes fused deposition modeling (FDM), also called fused filament fabrication (FFF), which is one of the most popular AM methods [32]. It is used for processing thermoplastic materials with high mechanical robustness [33,34] such as polylactic acid (PLA), acrylonitrile-butadiene-styrene (ABS), polystyrene (PS), polyethene (PE), polyethene terephthalate (PET), polycarbonate (PC), polycaprolactone (PCL), polyether ether ketone (PEEK), nylon, thermoplastic urethane (TPU), poly (vinyl alcohol) (PVA), and polyamides (PA). FDM allows the low-cost, low-volume production of parts with diverse geometries that are hard to achieve with casting and extrusion [32]. However, it is not a method free of drawbacks and challenges. Key parameters to consider include temperature control, deposition pattern, and layer thickness, which influence the final properties of FDM-printed parts [32]. FDM is particularly problematic for semi-crystalline polymers such as PEEK and PA because their high crystallinity frequently induces high shrinkage, interlayer delamination, and voids (pores) [33].

In this study, we comprehensively researched the preparation of raw CSs for surface modification by chemical methods for the preparation of a PA-12 composite for 3D printing by FDM. This work builds on our earlier experience [35,36]. Our main research motivation was to prepare a PCC for potential medical applications that is better than pure PA-12. An additional aspect is to have better resistance to SBF and to obtain a good polymer–ceramic interface. In the course of the work, CSs were thoroughly characterized after purification before surface modification, including their chemical and phase composition and grain size. Then, the surfaces of the cleaned cenospheres were chemically modified to improve the adhesion of cenospheres to PA-12. A two-step process was developed: (1) etching in Piranha solution and (2) silanization in 3-aminopropyltriethoxysilane (APTES). The CSs prepared in this way served as a filler for PA-12 and were used to prepare a composite for FDM printing. The obtained samples were subjected to soaking tests in artificial saliva for 14, 21, and 29 days in an autoclave under conditions simulating an oxidative environment. The obtained samples were subjected to mechanical tests. The control group consisted of samples not subjected to soaking tests. The importance of this research is that it shows the applicability of CSs in medical applications, but not limited to only that. Composites of this type are suitable for acoustic applications, energy storage, and structural applications [37,38,39]. The manner of their surface modification is also important as it helps to avoid phase separation [40]. These composites with these kinds of fillers are also part of sustainable technology [41,42].

## 2. Materials and Methods

### 2.1. Materials

#### 2.1.1. Cenospheres’ Preparation

Cenospheres (CSs) (origin: GRES-2 Powerplant, Kazakhstan) were selected as the composite filler. The preparation of the cenospheres was a multi-step process and included: (1) cleaning of CSs from post-industrial pollution, (2) removal of damaged cenospheres, (3) screening of cenospheres on a vibrating tower to obtain the desired grain fractions, (4) chemical surface modification by etching in Piranha solution, (5) chemical surface modification by APTES, and (6) thermal treatment. The preparation of the cenospheres was carried out based on previous investigations with alumina and zirconia surface modification [34,35]. The cenospheres were first cleaned of biological residues and sediments from the lagoons in which they were stored. For this purpose, sedimentation initially removed the sediments and damaged the cenospheres. The cenospheres were poured into a 1000 cm^3^ beaker with demineralized water and allowed to settle for 24 h to remove damaged ones. An ultrasonic washer (140 MHz, 1 h) was used for cleaning. The floating cenospheres were then poured from the solution into a separate beaker with a large magnetic stirrer and stirred in 2-propanol (CAS: 67-63-0, Acros Organics, Geel, Belgium). Iron-containing cenospheres and other magnetic particles adhered to a large magnetic dipole. Purified cenospheres were then poured from the solution into a crystallizer and dried (80 °C, 12 h). The thus-prepared cenospheres were fractionated on a laboratory shaker (Multiserw Morek LPzE-2e, Marcyporęba, Poland) with sieves certified at 45, 90, 150, and 212 μm (Multiserw Morek, Marcyporęba, Poland). The selected fractions were subjected to comprehensive physicochemical investigations, including BET, PSD, FTIR, XRD, XPS, and SEM. Based on the tests and the efficiency of the sieving process, the 90 μm fraction was chosen for the preparation of composite samples. The obtained cenospheres were subjected to a two-step chemical modification according to a process previously developed for alumina and zirconia [37,43].

**Figure 1:**
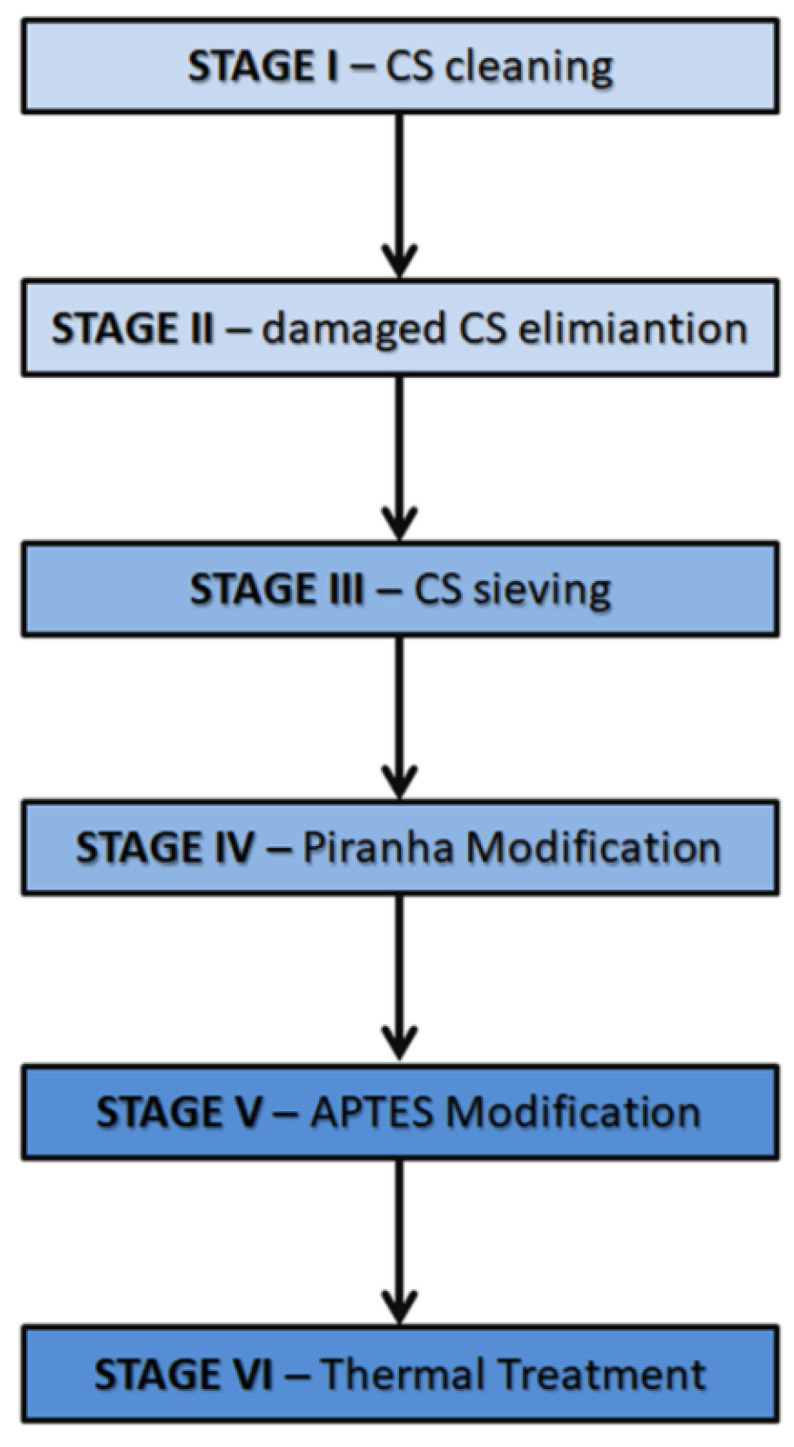
The purification and modification process of CSs used in this study.

In the first modification stage, CSs were subjected to etching modification in fresh, hot Piranha solution. The etching solution was prepared from H_2_SO_4_ (CAS: 7664-93-9, 95%, Acros Organics, Geel, Belgium) and H_2_O_2_ (CAS: 7722-84-1, 30%, STANLAB, Gliwice, Poland) in a volumetric ratio of 3:1. The powders were poured together with the Piranha solution into a round-bottomed flask and heated under reflux (100 °C, 15 min) with continuous stirring with a mechanical stirrer in a heating bowl (350 rpm). Then, both powders were filtered under vacuum with a water pump (washing 2 × 500 cm^3^). The solution pH was checked, and CSs were neutralized with ammonia water (CAS 1336-21-6, 25%, AVANTOR, Gliwice, Poland) with continuous stirring on a magnetic stirrer (200 rpm). In the second step, CSs were etched with APTES (3-aminopropyltriethoxysilane; CAS: 919-30-2, Acros Organics, Geel, Belgium). A Cp10% solution of APTES in 2-propanol (PrOH) (CAS: 67-63-0, Acros Organics) was used. The CSs in the APTES-PrOH solutions were stirred using a magnetic stirrer (30 °C, 24 h, 250 rpm). After mixing, the suspension was sonicated (*f* = 37 kHz, power 120%, 50 min, 30 °C, degas mode). The CSs were then filtered under reduced pressure with a water pump (2 × 500 cm^3^ wash). The CSs were then dried (forced air dryer, 12 h, 80 °C) and calcined in a muffle furnace (RENFERT Magma, Hilzingen, Germany) at 450 °C in an air atmosphere (temperature gradient 9°/min, isothermal holding for 2 h, cooling together with the furnace). The simplified purification and modification scheme is shown in Figure 1.

#### 2.1.2. Filament Preparation

**Figure 2:**
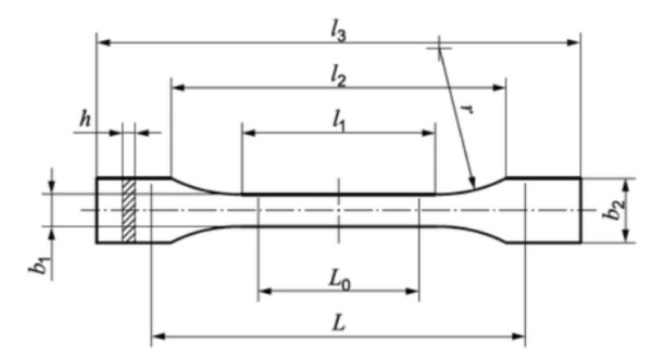
Sample type 1BA for strength tests.

The filament was prepared using a method similar to the one described by us in our earlier work [35]. To prepare the feedstock, a twin-screw extruder was used for the compounding, while a single-screw extruder was used for the filament preparation. To avoid hydrolysis, the PA-12 (VESTAMID PA12, Evonik, Essen, Germany) granulate was pre-dried at 50 °C for 10 h. The CSs were dried at 150 °C for 10 h. The molecular weight of the PA-12 ranged from 9100 to 16,600 g mol^−1^ [43]. An EBVP 25/44D extruder from O.M.C. SRL (Saronno, Italy) was used for compounding. The CS and polymer granules were dosed gravimetrically with a mass ratio of 20% CS and 80% PA-12. The CS content was determined experimentally based on trials. Contents greater than 20% caused delamination of the filament during extrusion and clogged the FDM printer head. A possible solution to this problem, already determined by us, was to use an FDM printing modification with a movable piston that regulated the pressure at the head outlet [39]. The extruder exit temperature of 260 °C was selected to be higher than the melting point of PA. After compounding, the polymer ceramic strand was cooled using a water bath and then granulated. A single-screw extruder from DR. COLLIN GmbH (Ebersberg, Germany) was used for shaping. The mass throughput was 3 kg/h at 14 rpm. After extrusion, the CS melt was pulled with a pull-off force, which depended on the crystallization degree of the carrier material. To set the pull-off force, filament diameters between 1.6 and 1.8 mm were used, which were recorded using a WIREMASTER and an ODAC 18 XY laser head from Zumbach (Orpund, Switzerland). The material used for comparison in batch 4 was a commercially available, white, 1.75 mm eco PLA filament from 3DJAKE (Niceshops GmbH, Paldau, Austria). The samples used for soaking and mechanical tests were prepared in the same manner as in our previous study [44]. Samples were prepared by FDM printing on another 3D printer (Double P255 by 3D Gence, Gliwice, Poland). Per the recommendations of ISO 527:2012 [A], type 1BA samples were prepared, which are preferred for machined specimens. The characteristic dimensions of the samples are shown in Figure 2 and Table 1. The 3D models were modeled in SolidWorks 2020 software. In order for the printer to read the file, the file was saved in the STL format, and the appropriate printing parameters were selected using 3D Gence Slicer 4.0. software (3D Gence, Gliwice, Poland).

The determination of all samples used in this study is presented below. Table 2 presents the determination of raw CSs, and Table 3 describes the samples used for the soaking and mechanical tests (samples containing CSs and reference samples of pure PA-12).

### 2.2. Methods

#### 2.2.1. XRD: X-ray Diffraction

Phase compositions were determined by an X-ray diffraction X’Pert Pro MPD (Panalytical, Almelo, The Netherlands), equipped with a copper anode lamp (λ_Kα_ = 0.154 nm) (Panalytical, Almelo, The Netherlands) as well as a PIXcel 3D detector (Panalytical, Almelo, The Netherlands) on the diffracted beam axis. The diffraction lines were recorded in the Bragg–Brentano geometry in the angular scope of 20–100° [2θ], with the step of 0.05° and the step time of 70 s.

#### 2.2.2. BET: Brunauer–Emmett–Teller

BET adsorption isotherms were performed on a Quantachrome Instruments iQ2 apparatus. The approximate pore shape and specific surface area were determined using BJH and de Boer models. Analyses were carried out under the following conditions: analysis time 1.73–2.33 h, final degassing temperature of the samples 3.6 h, and liquid nitrogen medium at 77.35 K.

#### 2.2.3. HP: Helium Pycnometry

HP was performed on an AccuPyc II apparatus (Micromeritics, Norcross, GA, USA). Analyses were conducted under the following conditions: chamber, 1 cm^3^; chamber helium filling pressure, 2.11 × 10^6^ Pa; measurement repeatability, ±0.01%; measuring accuracy, 0.03% of reading plus 0.03% of volume range sample chamber; and gas, pure helium for analysis. The number of measurements per sample was five.

#### 2.2.4. PSD: Particle Size Distribution

The particle size distribution (PSD) was measured using a laser particle sizer (Analysette 22, Fritsch GmbH, Diez, Germany). The dry powder samples were added to the 0.1% wt aqueous solution of tetrasodium pyrophosphate (NaPP). For each sample, five measurements were made to confirm the repeatability of the results.

#### 2.2.5. SEM/EDS: Scanning Electron Microscope/EDS Probe

A ZEISS SUPRA 35 scanning electron microscope (SEM) was used and equipped with an energy-dispersive X-ray spectrometer (EDX; UltraDry EDS Detector, thermo Fischer, Waltham, MA, USA), and Thermo Scientific™ Pathfinder™ X-ray Microanalysis software was used to determine the chemical composition of the analyzed samples. Detailed SEM images were taken with a TESCAN VEGA microscope (Brno, Czech Republic) using secondary electron (SE) and backscattered electron (BSE) modes. In addition, TESCAN firmware was used to determine the density differences in the materials.

#### 2.2.6. FTIR: Fourier-Transform Infrared Spectrometer

Fourier-transform infrared (FTIR) spectroscopy was used to analyze the characteristic functional groups in the samples using a Shimadzu IR Tracer-100 FTIR (Shimadzu, Kioto, Japan) spectrophotometer (Michelson interferometer; beam splitter: KBr germanium coated; light source: high-energy ceramics; detector: DLATGS detector) using a multi-reflection ATR attachment equipped with a diamond prism. Before analysis, the device was calibrated with a closed ATR attachment to record the background spectra. Then, the test samples were placed on a diamond, pressing against the prism with a dynamometric screw each time with the same force. To analyze and interpret the characteristic bands of the samples, the transmission spectra were recorded on a multi-reflection device. The analysis was performed automatically using the dedicated LabSolution IR software provided by the spectrometer manufacturer. Additionally, the recorded FTIR spectra were compared to the theoretical frequencies calculated with Gaussian 09 and GaussView 5.0. To minimize errors, 100 counts were performed with a resolution of 4 cm^−1^ for each analysis. The research was conducted from 4000–400 cm^−1^.

#### 2.2.7. Soaking Test

Samples from both composites were kept sealed in a high-pressure autoclave (Carl ROTH, Model-1) filled with artificial saliva (chemical composition in Table 4, according to EN ISO 10993-15:2000). The autoclaves were placed in a forced-air dryer (Binder) at 37 °C for 14, 21, and 29 days. After exposure to the artificial saliva, the samples were subjected to mechanical tests.

#### 2.2.8. Mechanical Testing

Static tensile tests were conducted following the recommendations of EN ISO 527:2012, with a tensile speed of 5 mm/min and using an MTS Criterion Model 45 with a 10 kN force sensor and MTS TestSuite software (MTS, Eden Praire, MN, USA). The separation between the grippers was 54 mm. These tests determined the maximum breaking force *F*_max_ [N], Young’s modulus *E* [GPa], elongation at break *A* [%], and tensile strength *R*_m_ [MPa]. Five replicates were performed for each sample.

Measurements of microhardness in the initial state and soaking samples were carried out using the instrumental method (Olivier and Phaar), which measures the resistance of a material to permanent deformation or damage. It is defined as the quotient of the maximum applied loading force and the projected contact area between the indenter and test sample [45,46]. The tests were carried out using the open platform equipped with a Micro-Combi-Tester by CSM Instruments (Micro-Combi-Tester, CSM instruments a company of Anton Paar, Peseux, Switzerland) using a Vickers indenter. The micromechanical properties were determined based on material deformation due to the indentation of the sample with a Vickers indenter to which a 100 mN maximal load was applied. The loading force and penetration depth of the indenter blade were recorded continuously during the entire cycle (loading and unloading). The loading and unloading rate was 200 mN/min, and the hold time of the sample at maximum load was 5 s. The microhardness result was the average of 10 longitudinal and transverse measurements across the entire surface area of composite samples in the initial state and after exposure to the artificial saliva. The values of the indentation hardness (*H_IT_*) and Vickers hardness (*HV_IT_*) were determined.

## 3. Results

**Figure 3:**
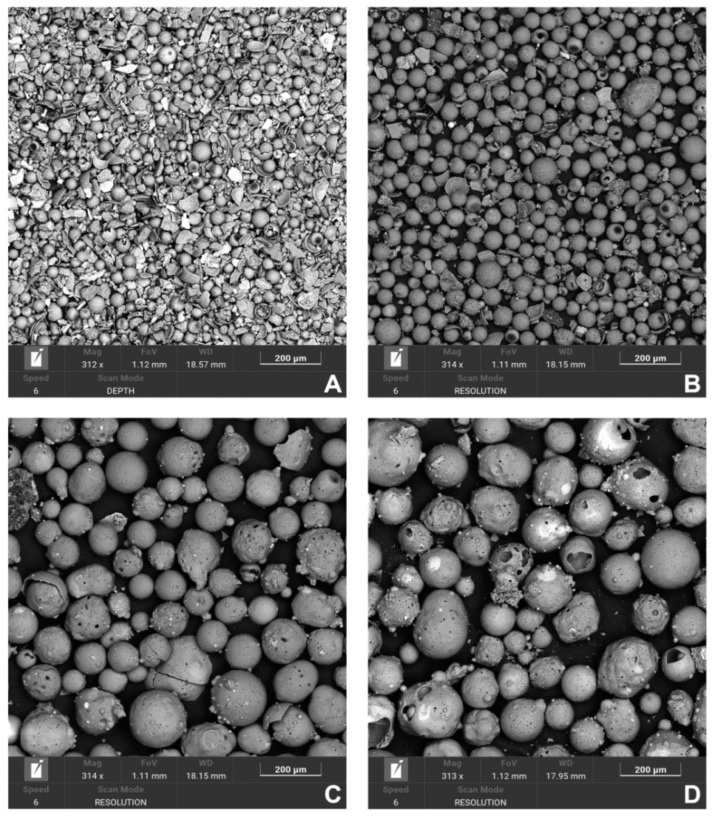
The obtained, purified cenospheres’ fractions (energy: 20 keV), mag. 300×: (**A**) F_45, (**B**) F_90, (**C**) F_150, and (**D**) F_212.

**Figure 4:**
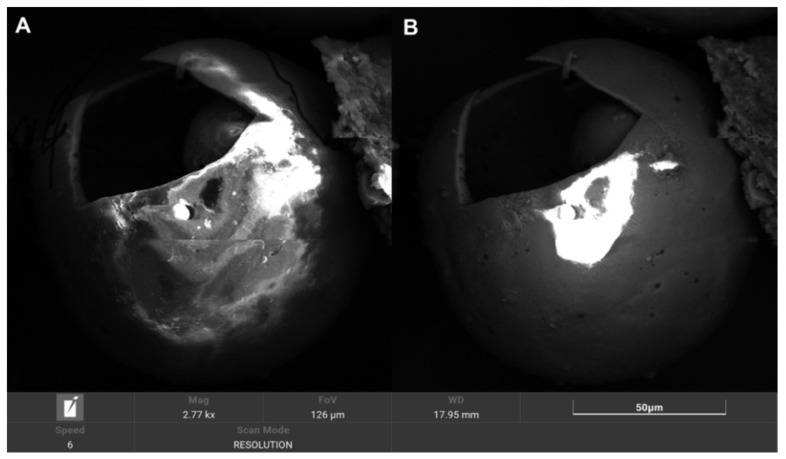
SEM microphotographs of the CS fraction F_212 (energy: 20 keV): (**A**) SE mode, (**B**) BSE mode.

**Figure 5:**
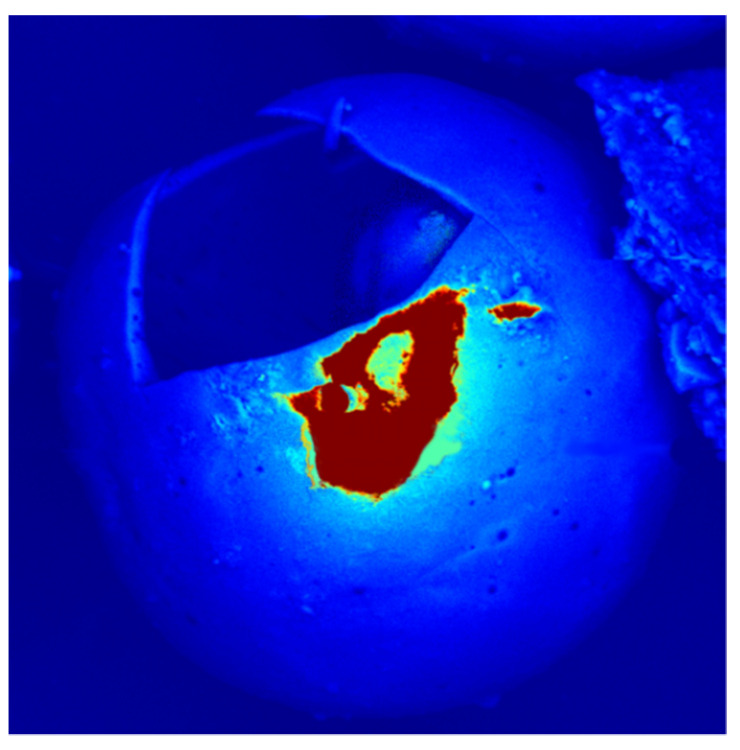
SEM microphotographs: CS fraction F_212 detail; we observed smaller CSs inside larger ones, mag. ×2770.

A summary of the results and a discussion of the physicochemical and mechanical testing and soaking tests are presented below. Based on the physicochemical measurements and the performance of the screening process, raw cenospheres were selected for the preparation of a composite using cenospheres with a 90 μm grain size. On average, 100 g of raw cenospheres were yielded by sieving at 15 g F_212, 31 g F_150, 48 F_90, and 6 g F_45. F_90 was also the fraction that was most homogeneous and had the fewest broken and damaged cenospheres; so, it was selected for further study. The figure below shows a comparison of the grain fractions (Figure 3). During the microscopic observations, we noticed that larger CSs, e.g., F_212 and F_150, sometimes contained even smaller microspheres (Figure 4 and Figure 5). This phenomenon has a “matryoshka-like” effect (Figure 5).

### 3.1. XRD

**Figure 6:**
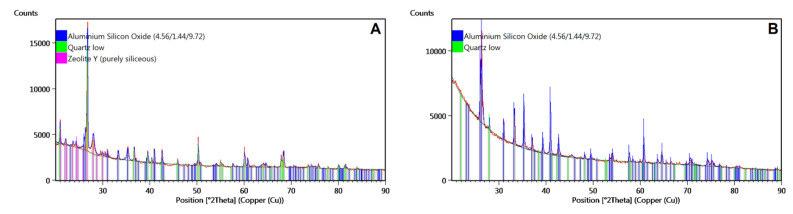

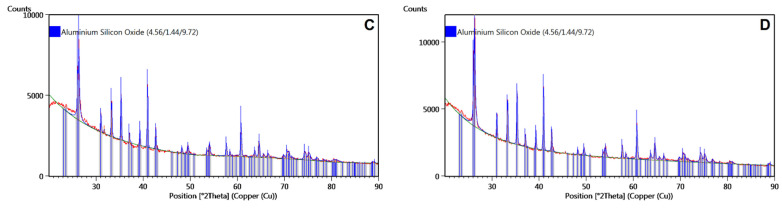
XRD patterns for raw CSs after purification: (**A**) F_45, (**B**) F_90, (**C**) F_150, (**D**) F_212.

XRD patterns of each CS sample after purification are shown below (Figure 6).

X-ray phase analysis (Figure 6C,D) showed that all samples contained aluminum silicon oxide Al_4.556_ O_9.722_ Si_1.444_ with an orthorhombic crystal structure (Inorganic Crystal Structure Database: 98-015-2980). All diffraction lines from the aluminum silicon oxide are indicated by blue lines. No differences were found for the F_150 and F_212 fractions (Figure 6C,D). Only the aluminum silicon oxide phase was identified in these samples. The diffractogram of F_45 also contained diffraction lines from quartz low (ICSD:98-016-2611), indicated by green lines, and Y zeolite (pure siliceous) (ICSD:98-005-5489), indicated by pink lines (Figure 6A). For fraction F_90, an intermediate composition between the previous three was noted; in addition to the mixture of aluminum and silicon oxide, quartz was also found.

### 3.2. BET

**Figure 7:**
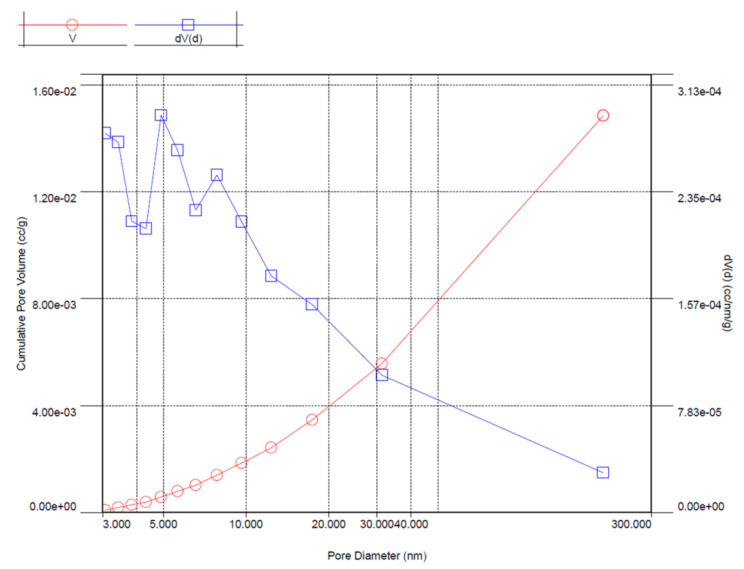
BJH–BET adsorption isotherms for F_45.

Representative results from the measurements of adsorption isotherms in liquid nitrogen are presented below (Figure 7). Table 5 shows the measured specific surface area (SSA), porosity, and pore distribution. The main objective of using BET was to determine the development of the specific surface area and the pore surface area (which made it possible to qualitatively determine in which samples there was ceramic scrap). As expected, a general regularity was noticed with the increase in degree of the specific surface area. SSA, as expected, was greatest for the CS_45 faction, which, additionally, had a lot of ceramic scraps. The lowest SSA was observed for CS_212. The rest of the data did not show a clear trend; however, for the cumulative pore volume, it was noted that the highest volume was obtained for F_45 and the lowest, for F_212.

### 3.3. HP

The results of real density measurements of the raw CSs are shown in the table below (Table 6).

For cenospheres’ fractions from F_90 to F_212, there was good correlation in the Miata: as the size increased, the density gradually decreased. For F_45 with the highest density, the result was related to the results from SEM, and the density measurements could not be taken literally. The density in these measurements depends on the significant presence of ceramic scrap.

### 3.4. PSD

**Figure 8:**
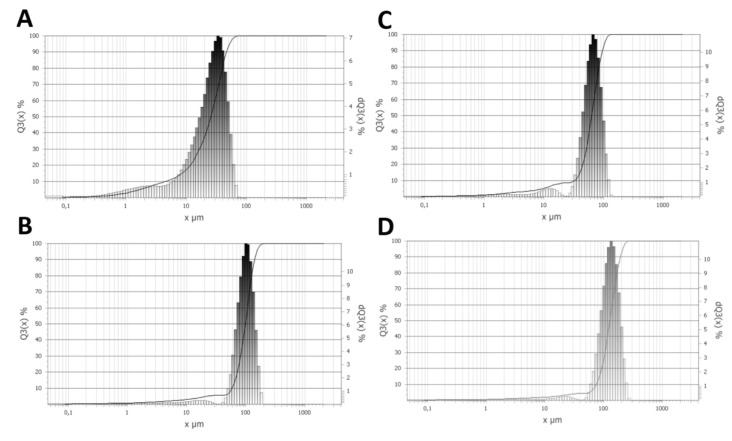
PSD results for raw CSs: (**A**) F_45, (**B**) CS—F_150, (**C**) CS—F _90, (**D**) CS—F_212.

Figure 8 shows that PSD was bimodal in all cases. However, some significant differences were observed. F_45 was characterized by bimodal values of 3 μm and 35 μm. Particles with a diameter smaller than 10 μm accounted for 17.5% of the sample’s volume. In the F_90, the bimodal nature of the distribution was more visible, and the particles were grouped into two populations separated by a gap between 21 μm and 23 μm. The modes moved toward higher sizes, namely, 12 μm and 70 μm. The particles with diameters smaller than 10 μm accounted for 5.2% of the sample’s volume. The PSD of the third sample had an identical shape to the second sample. Once again, the modal values and gap between the two populations of the particles moved to higher sizes. Here, the volume fraction of particles smaller than 10 μm was 2.9%. Finally, in the case of the fourth sample, the volume fraction of particles smaller than 10 μm was 1.9%. As for the previous samples, the modal values increased. With each sample, the maximum diameter increased. In the case of the first sample, there were no particles with a diameter larger than 80 μm; in the second case, 150 μm; in the third case, 200 μm; and in the fourth, case 250 μm.

### 3.5. SEM/EDS

**Figure 9:**
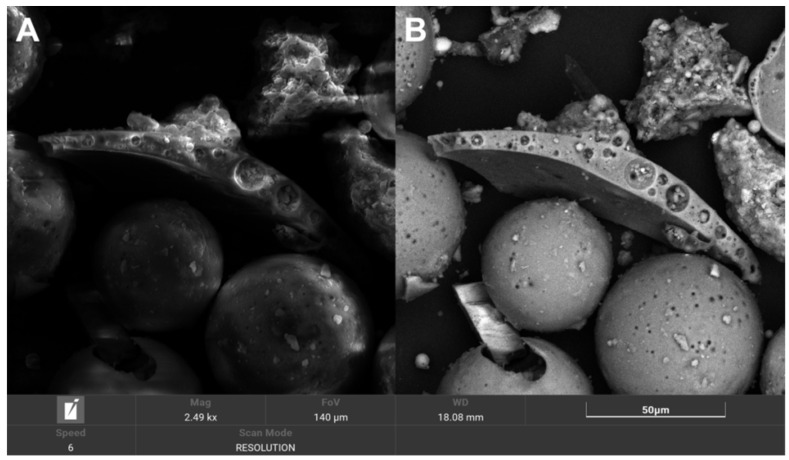
SEM microphotographs for CS F_90 fraction (energy: 20 keV): (**A**) SE mode, (**B**) BSE mode.

**Figure 10:**
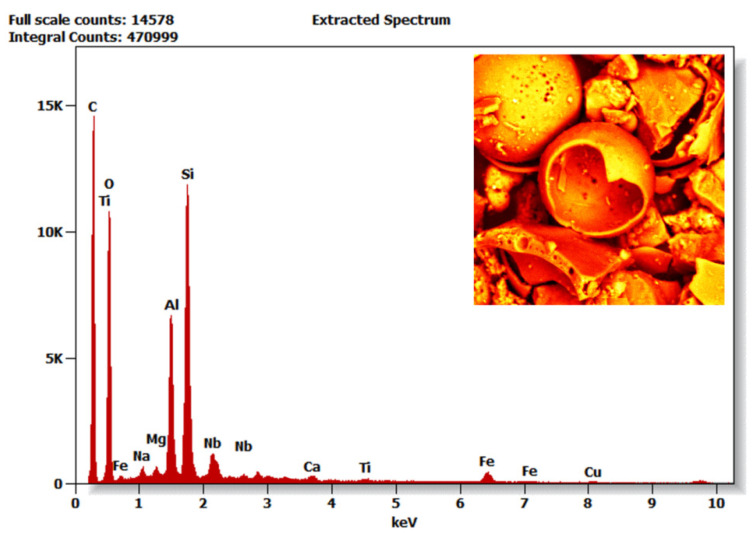
EDS spectra for CS F_45 fraction (energy: 20 keV).

SEM observations of the CS samples from all grain fractions and their chemical compositions are presented below. Figure 9 shows the detailed morphology of the F_90 fraction used to prepare the composite. Table 7 compares the chemical composition of all fractions referenced to fraction M_90; high levels of iron were observed in fraction F_45 (Figure 10).

### 3.6. FTIR

**Figure 11:**
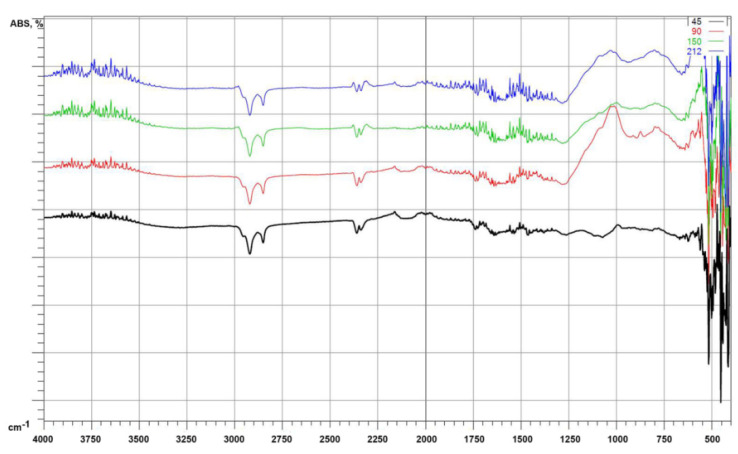
FTIR spectra for raw CSs: 45-F_45, 90-F_90, 150-F_150, and 212-F_212.

**Figure 12:**
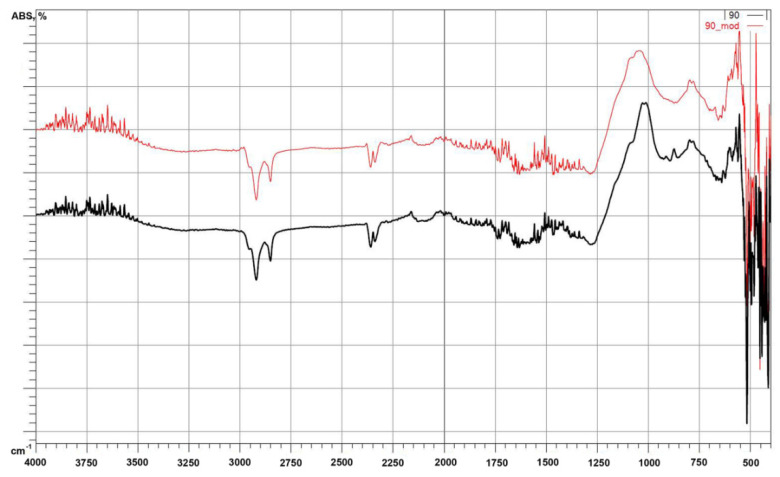
Comparison of FTIR spectra for 90-F_90 and 90_mod-M_90.

The figures below show the results of the measurements for the non-chemically modified microspheres (Figure 11). FTIR spectroscopy was performed to investigate the effect of silanization of the CSs. Figure 12 shows a comparison of the F_90 and M_90 fractions. In the FTIR spectra of the unmodified CSs, except for the F_90 fraction, no significant differences were observed. However, for the F_90 fraction at wave number values equal to 800, 850, and 900 cm^−1^ there were differences. Differences were also observed for the F_90 fraction after modification: for M_90, the peaks in the spectrum of F_90 at 850 cm^−1^ disappeared. The bands between 3400 cm^−1^ and 1550 cm^−1^ were attributed to the stretching vibration and bending vibration of -OH groups of H_2_O molecules, respectively, which indicates the presence of a small amount of molecular water in the samples [47]. This is especially visible in the range of 1550–1650 cm^−1^. The bands between 1070 and 1100 cm^−1^ were attributed to the asymmetric stretching vibrations of Si-O(Si). In addition, a broad band around 1100 cm^−1^ was due to pure silicon. As the Si/Al ratio increased (as for the F_90 fraction), the band had a higher intensity at smaller wavenumbers due to the substitution of aluminum atoms for Si at the tetrahedral position [47,48]. The bands near 500–580 cm^−1^ were due to Si-O-Al vibrations in the CSs, which formed from the structural rearrangement of the Si-O-Al vibrations [47,48]. The last bands identified for the unmodified CSs between 500 and 450 cm^−1^ were associated with the bending vibrations of O-Si-O in the silicate tetrahedra. By conducting a comparison for the modified sample, the spectrum over the whole range showed slight differences. Different bands were observed for M_90 at 450, 470, 600, 875, and 1050 cm^−1^. The bands at 450 and 470 cm^−1^ were connected with the bending vibrations of O-Si-O in the silicate tetrahedral [47]. The vibrations at 600 cm^−1^ corresponded to N-O deformation. At 875 cm^−1^, the band corresponded to C-N stretching, and the band at 1050 cm^−1^ corresponded to N-O stretching.

### 3.7. Mechanical Testing

**Figure 13:**
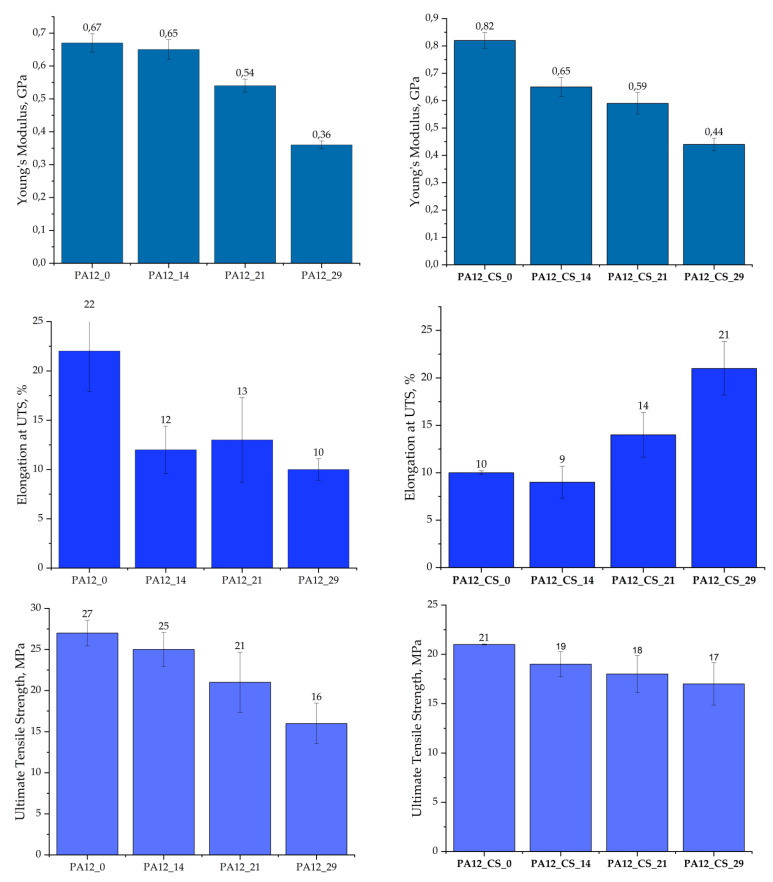
Young’s modulus, elongation at ultimate tensile strength, and ultimate tensile strength of the PA12 and PA12_CS in the initial state and after soaking in artificial saliva (SBF).

The tensile test results of PA12 and PA12_CS in the initial state and after exposure to artificial saliva after 14 days, 21 days, and 29 days are shown in Figure 13. The ultimate tensile strength in the initial state was 27 MPa (±1.57 MPa) for PA12 and 21 MPa (±0.05 MPa) for PA12_CS. After soaking in the artificial saliva, the tensile strength decreased with the time for PA12, while, for PA12_CS, no significant change was observed.

The microhardness results for all samples are presented in Table 8. Based on the data, it can be concluded that, in the initial state, PA12_0 (*HV_IT_* = 15) was characterized by a lower hardness compared to the initial state of PA12_CS_0 (*HV_IT_* = 29). Soaking the PA12 samples in the artificial saliva for 14 days increased the hardness, while, for 21 days, it decreased and, for 29 days, the hardness significantly decreased. Instead, the PA12-CS samples showed a two-fold decrease in hardness after 14 days, then a slight decrease after 21 days and 29 days. The measurements were taken at different areas of the samples, and no differences were found across the entire area.

## 4. Discussion

**Figure 14:**
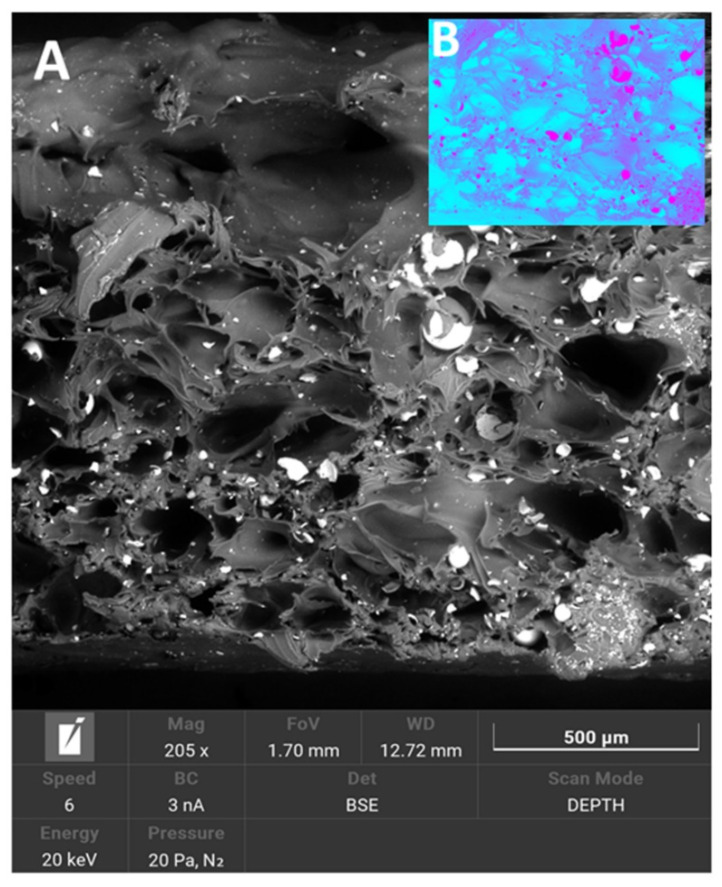
SEM microphotographs: breakthrough PA-12_CS samples: (**A**) SEM image, (**B**) image showing the marked inhomogeneous distribution of CSs in the matrix (concentrations of saturated pink color).

Specimens were prepared from raw CSs and PA-12, with which we previously attempted to prepare a composite [44]. The procedure and conclusions from the preparation of the composite with PA-12 are basically in line with the observations for ZrO_2_ and Al_2_O_3_, which were used as ceramic fillers in our previous study [41]. As far as the treatment itself and the preparation of CS are concerned, significant problems were observed when attempting to eliminate biological wastes from the samples. The presence of iron, due to its ferromagnetic nature, is undesirable in this case. We attempted to remove black particles from CSs containing iron compounds from the material using a magnetic field from an agitator dipole. However, this process was only effective for CSs with a size greater than 45 μm. For smaller sizes, there was a significant proportion of iron in the chemical composition (Table 7). Correlating these observations with the morphology, we found that the presence of iron was in the form of a ceramic, sharp-edged scrap, which was not observed in the other samples (Figure 3A–D). The main problem observed during the preparation of the PA-12–CS composite was that the ceramic filler mixed very poorly with the polymer matrix. Compared with previous results for ZrO_2_ and Al_2_O_2_ [44], where it was possible to use 30 wt.% ceramic powder and 70 wt.% polymer, only 20 wt.% was possible. Larger amounts no longer mixed with PA-12, and the filament cracked during extrusion. Another peculiarity was that CSs at levels above 20 wt.% floated on the surface in the extruder tank and were no longer wetted by the polymer. This was seen in fracture images, where the CSs were unevenly distributed, and represented a less satisfactory result relative to our previous composites (Figure 14) [44]).

Other authors have shown variations in the mass percentage of CSs [49,50], which can range from 3 to 15 wt.% in composites prepared using injection molding or simple molding [51,52]. The dispersion of CSs has not been analyzed before; however, the problem of CSs binding with a polymer matrix has been pointed out, and the need for a coupling agent has been demonstrated [47]. We can guess that this is mainly due to problems with wetting of the CSs’ surface by the liquid polymer and poor adhesion of the ceramic to the polymer.

Based on the BET and PSD measurements, the main characteristics of the shape and the size distribution of the CSs and their specific surface area were determined. As mentioned previously, the PSD was always bimodal. Correlating these results with the SEM observations, one can clearly state that the smaller peak belonged to the ceramic scrap (broken CSs and compounds containing Fe_2_O_3_). The presence of ceramic scrap would adversely affect the composite structure because the scrap would sediment in the dies during extrusion, increasing the density and, thus, the weight of the composite, and possibly cause phase separation. Therefore, CSs with scrap were excluded from further investigations. Relating these results to the adsorption isotherms, F_45 has the highest surface area and pore volume (Table 5). The surface area and pore volume were almost exactly inversely proportional to the size of the CSs.

As far as the analysis of the phase composition of raw CSs is concerned, no major differences were noticed between the samples. This is also consistent with general information about CSs [19]. The chemical and phase composition of raw CSs strongly depends on the origin of the extracted stone coal and on the way it was burned (mainly the degree of coal grinding before burning in a furnace) [19]. Therefore, it can be concluded that it is not possible to generalize the information on phase composition and introduce a single standardization for all CS samples. It follows that raw CSs should be tested each time before preparing a new material, such as using XRD measurements. The specific phase composition corresponds to generally known data for CSs: phases can be separated into mullite, cristobalite-quartz, K-feldspars, acid plagioclases, and magnetite [19]. It follows that CSs are infused with quartz, lime, and periclase, which compose the skeleton of the cenospheres [19]. The rest are amorphous fillings. For CSs below 150 μm, the potassium and calcium levels decreased because these cenospheres were highly crystalline. As the diameter increased, the proportion of glasses increased, and CSs were predominantly amorphous [19]. In contrast, there was no major correlation in the chemical composition related to the occurrence of carbon (Table 7). After cleaning the post-processing residues (large ash grains, organic remains, sediments, and stones), for fractions F_90 and F_45, there were differences compared with the other samples. Both fractions contained niobium, and F_45 contained the highest carbon content and, surprisingly, iron (Table 7). The chemical composition corresponded to that as reported by Ranjbar and Kuenzel and Agrawal and Wanjari [19,53]. According to these observations, the CSs were mainly composed of Al and Si, predicted theoretically using the equation proposed by Fomenko et al. [54]:

SiO_2_ ➔ 4.34[Al_2_O_3_] − 0.08[Al_2_O_3_]^2^ (1)

This was consistent with our analyses. However, as noted by Agrawal and Wan-Jari there should be no carbon in CSs after purification [53]. This conclusion can also be drawn by analyzing the work of Ranjbar and Kuenzel [19]. It can be concluded that carbon originates from process impurities, which (especially for CS samples smaller than 56 μm) are very difficult to remove with our proposed methods. In this case, the use of surface-active agents or ionic liquids should be considered. Two forms of Fe^3+^ have been found: single ions and nanoparticles with a diameter of 3 to 5 nm that consist of a superparamagnetic phase with a spinel structure whose sublattices are diamagnetically diluted with Mg^2+^ and Al^3+^ ions [19]. Magnetic cenospheres have heterogeneous regions of ferrospinels on their outer surface. Upon increasing the iron concentration, the crystallite size of the ferrospinel phase increased, whereas the degree of substitution of iron (magnesium and aluminum) decreased [19]. In observations, this was manifested by the presence of distinct black spots on the surface of the cenospheres, which would also confirm which cenospheres were removed during clearing before surface modification. In magnetic particles with porous shells and similar average diameters, an increase in the Fe_2_O_3_ content in the range of 4–21 wt.% led to an increase in the content of spotty spheres with a non-uniform surface in the range of 13–81 vol.% and an increase in the content of dark globules with a smooth surface up to 26 vol.% [19]. Cenospheres are by-products of coal combustion and have alkaline chemical compositions similar to fly ash. As observed, depending on the chemical composition of fly ash and cenospheres, they can be classified into subgroups: sialic, ferrocalsialic, ferrosialic, calsialic, ferrocalcic, and calcic [19]. Additionally, depending on this, CSs also have different sizes and, thus, chemical compositions. As levels of iron compounds increased, larger CSs were obtained [19]. Larger CSs also have thinner shells [19]. As the cenospheres’ sizes increased, their spheres were more open-worked and their shells more susceptible to damage (Figure 3C,D). These observations can be related to density measurements: the narrowest CSs also have the lowest density (Table 6). Perhaps this is what confirms the fact that the ceramic scrap for F_45 comes from the CSs of larger sizes we observed and contains precisely elevated amounts of iron compounds. Another tendency for CSs observed by other authors is that increasing the Al_2_O_3_/SiO_2_ ratio promotes increasing the particle size [19]. However, there is certainly a correlation between the presence of ceramic scrap and a significant increase in density for F_45 (Table 5). However, the authors pointed out that this subject is still poorly understood. There have been other completely contradictory reports; it should be approached with caution and additional studies should be performed on a larger number of CSs from different sources [19]. The differences in chemical composition for bulk and CSs’ surface measured by EDS seem to confirm the regularities reported by other authors [19]. Due to the complex structure of CSs, the solid crystal skeleton is immersed in amorphous phases and various crystalline phases in “spot-like” structures on the surface [19]. The distribution of these fractions is random.

**Figure 15:**
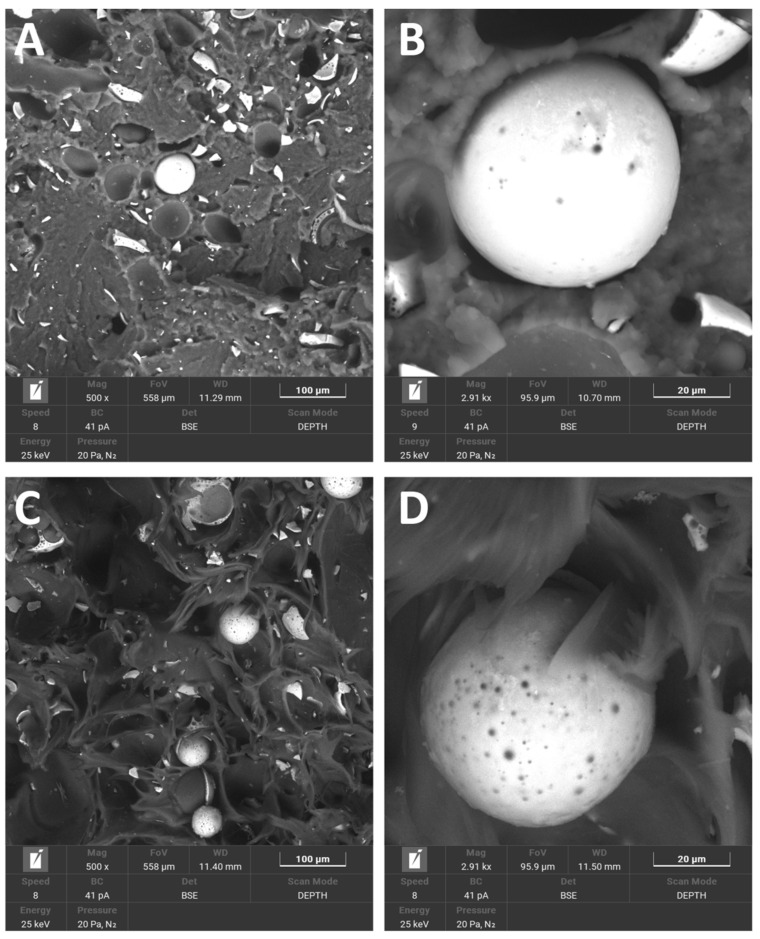
SEM microphotographs of breakthrough PA-12_CS samples: (**A**) CSs without a surface modification, (**B**) detail: surface of unmodified CSs, (**C**) CSs with surface modification, (**D**) detail: surface-modified CSs.

These observations coincided with our observations of the CSs we used as raw materials. As for the effect of the surface modification of CSs, it was confirmed by EDS; the data are included in Table 7. Analyzing the data for CS F_90 and M_90, the presence of nitrogen was noticed for the modified samples, as well as an increase in the proportion of silicon and a decrease in the concentration of aluminum and niobium. Changes in the chemical composition after modification were also confirmed by FTIR. The presence of additional silicon from the APTES pot was confirmed by the bands between 1070 and 1100 cm^−1^, which are characteristic of the stretching vibrations of Si-O(Si) [55]. The presence of nitrogen was confirmed by the peak at 1050 cm^−1^. An interesting thing that was observed was the occurrence of the correlation between the surface modification with APTES, the surface etching with Piranha solution, and the adhesion of CS to PA-12. Based on SEM observations and chemical composition correlations from EDS and verification with FTIR, etched and modified APTES CSs had stronger bonds with PA-12. Figure 15 shows a comparison of PA-12 CSs in two variants: (A) and (B) CSs without surface modification and (C) and (D) CSs after the whole two-step modification process. For the composite where CSs were modified, the polymer clearly “stuck” to the ceramics and, at the breakthrough after stretching, CSs did not fall out. There were no pop-outs; only the CSs were crushed but remained in the matrix. Additionally, the Piranha solution formed holes on the surface from etching. For a composite where the CSs were not modified, there was a clear lack of adhesion except for mechanical “wedging” into the matrix. On the sphere surfaces, there were only single holes, resulting from the nature of the material. Numerous pop-outs at the breakthrough confirmed poor adhesion, and the entire CS fell out during mechanical testing.

The samples after the soaking tests were subjected to mechanical tests to confirm the influence of SBF on potential aging under real conditions. Regarding the effect of the soaking time on pure PA-12, it was noted that, upon increasing the soaking time, the hardness decreased from 15 *HV_IT_* for PA12_0 to 0.4 for PA12_29. A similar trend was observed for microhardness (Table 8); however, a sharp decrease in hardness occurred only after 21 days of exposure in SBF. For PA12_CS, a significant improvement in hardness and SBF resistance was observed over the whole time from 14 to 29 days (Table 8). The drop in hardness was milder, only 2 HV from days 14 to 29, which showed how CSs increased the stability of our material in SBF. Relating our results to our previous studies with PA12_ZrO_2_, for PA12_Al_2_O_3,_ we observed the following phenomena. Compared with previous studies on zirconia and alumina, the present samples from the CSs had a higher initial hardness (PA12_ZrO_2_—11 HV, PA12_Al_2_O_3_—17 HV) [42]. On the other hand, the hardness values after 29 days in SBF were PA12_ZrO_2_, 19 HV; PA12_ZrO_2_, 19 HV; and PA12_Al_2_O_3_, 12 HV [44]. Although the values of 14 and 21 HV for PA12_CS were lower, the material behaved more predictably, and the decrease in the hardness was almost linear. Unfortunately, we have not found any studies that used a similar polymer matrix as ceramic oxides or studies in SBF; so, we have no way to refer to other authors on this topic. In terms of Young’s modulus, elongation, and ultimate tensile strength, however, the following pattern was observed (Figure 13). For both pure PA12 and PA12_CS, a decrease in modulus *E* was recorded during the soaking test. However, PA_CS had a higher *E* at the beginning and end than pure PA12. Importantly, after 29 days, 0.44 GPa was recorded for PA12_CS and 0.36 GPa was recorded for pure PA12. For PA12_CS, the values at each soaking time were higher than for PA12_ ZrO_2_ and PA12_ Al_2_O_3_ [43]. Generally relating the results to our previous work and future investigations, we found the following trends [44]:

- Something that draws our great attention is the different values for the strength parameters for pure PA-12. There are two reasons for this: the study was performed on two different mechanical testing machines (which did not have that much influence) and PA-12 granulates were probably more dampened. This conclusion can be drawn from the fact that the strength values, E modulus, UTS, and elongation changed significantly (elongation, in particular, from 15 to 22%). This led to the conclusion that the polymer was insufficiently dried and had residual moisture, which significantly worsened its mechanical properties; hence, this preparation error negatively affected the properties of the overall composite.
- Compared to zirconia and alumina composites, PA-CS has different properties, i.e., before soaking, it had lower mechanical properties, lower E modulus, and lower UTS. However, for composites subjected to soaking, these values changed. PA-CS lost less in comparison to zirconia- and alumina-reinforced PA-12. These differences are due to the nature of ceramic filler: CS as a filler acted similarly to a ball bearing and caused sliding of the material, causing it to display more elastic behavior.
- The prepared material, before being subjected to numerical tests, must be additionally subjected to further technological tests: determination of additional parameters of granulate injection, time and temperatures of granulate drying, conducting 3D printing in a greater number of variable parameters, characteristics for the arrangement of the filament in the x, y, and z planes, and, what seems to be the most important, conducting long-term tests of artificial aging on new kinetic models, taking into account the time, temperature, pressure, and variable simulation fluid in which the processes are conducted.

## 5. Conclusions

This study aimed to purify CSs and modify them with APTES and obtain a medical-grade PA-12 composite. We noted the following key results and observations:

1. CSs are a noteworthy material, which can be suitable for various mechanical and industrial applications. A lot of attention should be devoted to the removal of ceramic scrap, which is responsible for the presence of undesirable inorganic compounds.
2. EDS surface modification results confirmed that APTES applied additional nitrogen and silicon compounds.
3. The initial problem with raw CSs was that CSs float on the surface of molten PA-12, mix poorly, and have a generally non-uniform distribution in the polymer matrix. For modified CSs, this problem was generally not present; however, more time should be spent on the development of a mixing technology for these types of fillers. With the current state of knowledge, it seems that a maximum of 20%wt CSs can be added as a filler.
4. As far as the improvement of the mechanical properties of PA-12 after the addition of CSs is concerned, the matter is debatable and requires further work. In comparison with the earlier composites with zirconia and alumina, PA_CSs have lower initial mechanical properties, while the samples behave better after soaking. In addition, an important conclusion regarding the storage of granulate PA-12 was that improper drying adversely affected the mechanical properties of the entire composite.

## Figures and Tables

**Table 1:** Sample dimensions.

Dimensions of the Sample	Dimensions, mm
***l*_3_**—overall length	75
***l*_1_**—length of narrow parallel-sided portion	30.5
***r***—radius	37
***l*_2_**—distance between broad parallel-sided portions	57.5
***b*_2_**—width at ends	10
***b*_1_**—width of narrow portion	5
***h***—thickness	2.35
***L*_0_**—gauge length	25
***L***—initial distance between grips	54

**Table 2:** Sample descriptions.

**Sample, Fraction**	0–45 μm	45–90 μm	90–150 μm	150–212 μm	Modified 45–90 μm
**Description**	F_45	F_90	F_150	F_212	M_90

**Table 3:** Descriptions of the samples used for soaking and mechanical tests.

Soaking Time, Days	0	14	21	29
**PA-12**	PA12_0	PA12_14	PA12_21	PA12_29
**PA-12_CS**	PA12_CS_0	PA12_CS_14	PA12_CS_21	PA12_CS_29

**Table 4:** SBF: artificial saliva chemical composition.

Compound	Na_2_HPO_4_	NaCl	KSCN	KH_2_PO_4_	NaHCO_3_	KCl
**Concentration, g/L**	0.260	0.700	0.330	0.200	1.500	1.200

**Table 5:** BET data for all CS fractions.

BET–BJH Characteristics	Surface Area, m^2^g^−1^	Pore Volume, cm^3^g^−1^	Pore Diameter, nm
CS Fractions			
F_45	2.146	0.015	4.906
F_90	0.696	0.006	3.427
F_150	0.403	0.008	6.585
F_212	0.367	0.005	3.428

**Table 6:** Real density measurement CS data.

Sample	Density *, g cm^−3^
**F_45**	1.682
**F_90**	0.84
**F_150**	0.83
**F_212**	0.79

* average of five measurements.

**Table 7:** Comparison of the chemical composition of all CSs’ fractions.

Composition, wt.%	C	O	Na	Mg	Al	Si	Ca	Ti	Mn	Fe	Nb	N
Fraction												
**F_45**	8.1	20.1	0.3	0.3	4.8	15.7	-	-	-	42.7	6.9	-
**F_90**	2.2	48.8	0.2	-	20.8	23.6	-	0.8	-	-	3.5	-
**F_150**	2.7	41.8	-	26.9	28.6	-	-	-	-	-	-	-
**F_212**	5.6	38.8	0.6	0.5	7.6	41.3	1.1	1.0	3.4	-	-	-
**M_90**	2.2	47.2	0.9	-	9.6	31.5	-	-	-	-	2.2	3.7

**Table 8:** Microhardness results of tested samples.

	PA12_0	PA12_14	PA12_21	PA12_29	PA12_CS_0	PA12_CS_14	PA12_CS_21	PA12_CS_29
Vickers hardness, *HV_IT_*	15 ± 6	20 ± 7	10 ± 2	0.4 ± 0.1	29 ± 4	15 ± 1	14 ± 2	13 ± 2
Microhardness *H_IT_*, MPa	163 ± 20	210 ± 30	110 ± 22	4 ± 1	302 ± 25	164 ± 7	149± 9	135 ± 11

## Data Availability

Not applicable.
